# Long‐anchoring Stent Method as a Rescue Technique for Early Dysfunction of Covered Self‐Expandable Metal Stents in Distal Malignant Biliary Obstruction: A Retrospective Case Series

**DOI:** 10.1002/deo2.70394

**Published:** 2026-09-03

**Authors:** Takumi Kinomoto, Yugo Kai, Minoru Shigekawa, Kenji Ikezawa, Takuo Yamai, Hiroki Takiyama, Taku Miyanaga, Takanori Masumoto, Keita Sekiya, Jun Murata, Makiko Urabe, Ryoji Takada, Kaori Mukai, Tasuku Nakabori, Kazuyoshi Ohkawa

**Affiliations:** ^1^ Department of Hepatobiliary and Pancreatic Oncology Osaka International Cancer Institute Osaka Japan; ^2^ Department of Gastroenterology and Hepatology Kansai Rosai Hospital Amagasaki Japan; ^3^ Department of Gastroenterology Osaka General Medical Center Osaka Japan

**Keywords:** anchoring stent, biliary drainage, distal malignant biliary obstruction, self‐expandable metal stents, stent dysfunction

## Abstract

**Objective:**

Covered self‐expandable metal stents (SEMS) are widely used for treating distal malignant biliary obstruction (MBO). Severe SEMS angulation can cause stent dysfunction. Placement of a long double‐pigtail plastic stent inside the SEMS (the “long‐anchoring stent method”) may straighten the angulated SEMS, thereby achieving effective biliary drainage. This study aimed to report the clinical outcomes of this novel method.

**Methods:**

We retrospectively analyzed 15 patients with distal MBO who underwent initial SEMS placement, subsequently experienced clinical failure or early recurrent biliary obstruction (RBO) within 90 days of initial placement, and underwent reintervention with the long‐anchoring stent method between April 2020 and March 2025.

**Results:**

The median follow‐up period was 307 days after the anchoring procedure. For the primary outcome, clinical success was achieved in all 5 patients with initial SEMS clinical failure and in all 15 patients overall. The median angle of the SEMS significantly straightened from 128.3° with the initial SEMS alone to 151.5° after anchoring stent placement (*p* < 0.001). As an exploratory analysis of the 10 patients with early RBO, the median time to RBO increased from 21 days after initial SEMS placement to 148 days after anchoring procedure. No severe procedure‐related adverse events occurred.

**Conclusion:**

The long‐anchoring stent method is a simple, safe, and viable strategy for rescuing early dysfunction of covered SEMS, possibly in part through straightening of the SEMS angulation.

**Trial Registration:**

N/A.

## 1 Introduction

Covered self‐expandable metal stents (SEMS) are widely used for treating distal malignant biliary obstruction (MBO) [1, 2] as they provide longer patency than plastic stents [3]. An additional advantage is that they can be removed or exchanged if necessary [4]. However, stent dysfunction, which includes clinical failure and recurrent biliary obstruction (RBO), remains a common clinical problem [5]. Early stent dysfunction often requires unplanned hospitalization for conditions such as cholangitis, leading to the delay or interruption of cancer therapy [6]. Therefore, reducing the risk of early stent dysfunction remains an important clinical challenge. Furthermore, the prevention of procedure‐related adverse events, including cholecystitis and pancreatitis, is another major concern [7]. To address these issues, SEMS designs with low axial force and high radial force are considered desirable, as they may help reduce the risk of RBO and complications [8, 9]. However, despite the use of these stents, stent dysfunction still occurs in clinical practice. Strong angulation of SEMS, particularly those with low axial forces, may contribute to RBO by impairing bile flow [10, 11]. In addition, patients with duodenal strictures or tumor invasion have a high risk of stent dysfunction associated with duodenobiliary reflux [12, 13].

To overcome these limitations, we propose a novel technique, the “long‐anchoring stent method.” Although anchoring double‐pigtail plastic stents have previously been used to prevent migration of covered SEMS [14, 15, 16], we hypothesized that they could also correct stent angulation. In this method, a double‐pigtail plastic stent is deployed through a strongly angulated SEMS, with its distal end positioned further into the duodenum. This approach is expected to straighten the SEMS by leveraging the axial force of the plastic stent and directing the distal end of the SEMS toward the anal side, improve bile flow, and reduce the risk of early stent dysfunction.

This study aimed to evaluate the long‐anchoring stent method as a rescue option for achieving effective drainage in patients with early dysfunction of the initial SEMS placement. Furthermore, changes in SEMS angulation were analyzed to clarify the underlying mechanism of this method.

## 2 Methods

### 2.1 Study Design and Patients

This retrospective study was conducted at the Osaka International Cancer Institute, Japan. The study included 15 patients with distal MBO who underwent a covered SEMS placement, subsequently experienced clinical failure or early RBO within 90 days, and received a long‐anchoring stent method as a reintervention between April 2020 and March 2025. Patients with hilar biliary strictures, benign distal biliary strictures, or postoperative complications were excluded. A history of prior plastic stent placement or biliary drainage did not preclude study eligibility.

### 2.2 Procedures

All endoscopic retrograde cholangiopancreatography (ERCP) procedures were performed using a side‐viewing duodenoscope (JF260V, TJF260V; Olympus, Tokyo, Japan). A covered SEMS was initially placed in patients diagnosed with distal MBO. In cases where clinical failure or RBO occurred within 90 days, the cause of occlusion was assessed through imaging studies, including computed tomography and subsequent endoscopic examination. After exchanging the initial SEMS or performing intraluminal cleaning, an anchoring double‐pigtail plastic stent (as a long‐anchoring stent) was placed through the SEMS in cases where the cause of clinical failure or RBO was suspected to be food impaction, sludge formation, or bile duct kinking. Patients with conventional anchoring stents, in which anchoring stents were placed to prevent the migration of newly placed SEMS, were excluded.

**FIGURE 1:**
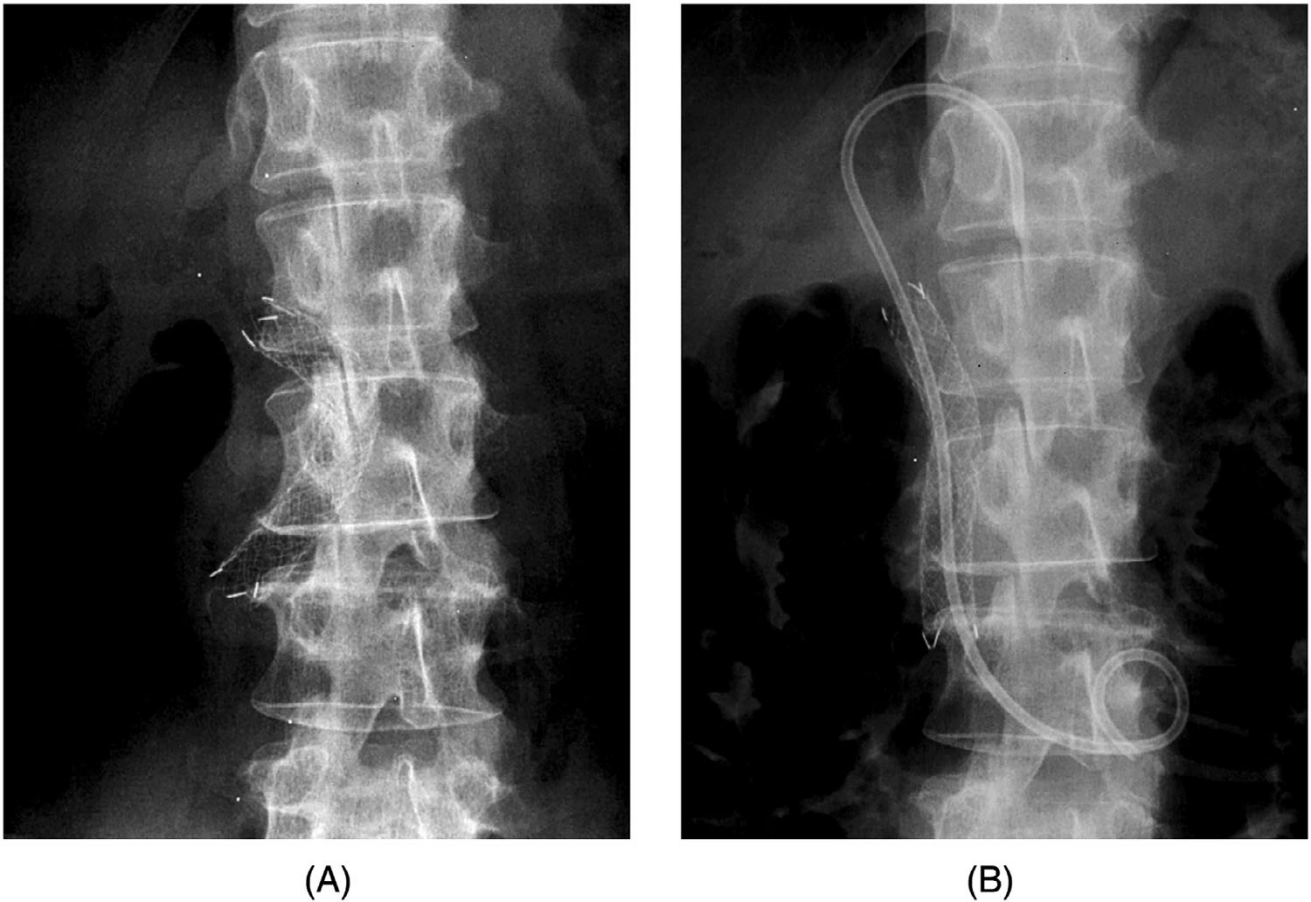
Fluoroscopic images of the long‐anchoring stent method: (A) initially placed self‐expandable metal stent (SEMS) was severely angulated; (B) the SEMS was not exchanged, and a double‐pigtail plastic stent was placed into the SEMS. The distal end of the plastic stent reached the horizontal part of the duodenum.

The anchoring plastic stent was inserted into the SEMS over a guidewire. Its proximal end was positioned in an intrahepatic duct branch or the common bile duct, and its distal end was extended beyond the distal end of the SEMS into the duodenum, ideally reaching the horizontal portion of the duodenum (Figure 1). The covered SEMS used in this study included Niti‐S ComVi stent (Taewoong Medical, Seoul, South Korea), BONASTENT (Standard Sci‐Tech, Seoul, South Korea), HANARO Stent (M.I. Tech, Pyeongtaek, South Korea), and WallFlex (Boston Scientific, Marlborough, USA). The anchoring plastic stents comprised double‐pigtail plastic stents (Medi‐globe, Achenmuhle, Germany) and Through&Pass (Gadelius Medical, Tokyo, Japan). The choice of plastic stent and SEMS type, as well as the decision to replace the existing SEMS or place the anchoring stent within it, was left to the discretion of the endoscopist.

### 2.3 Outcomes and Definitions

**FIGURE 2:**
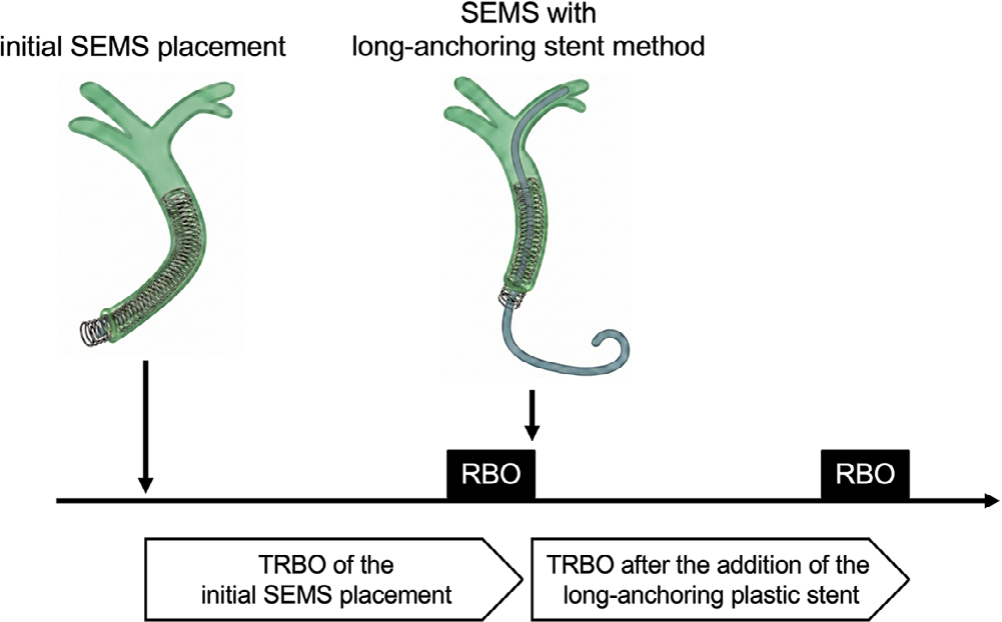
Clinical course of patients in this cohort. SEMS, self‐expandable metal stent; RBO, recurrent biliary obstruction; TRBO, time to recurrent biliary obstruction.

**FIGURE 3:**
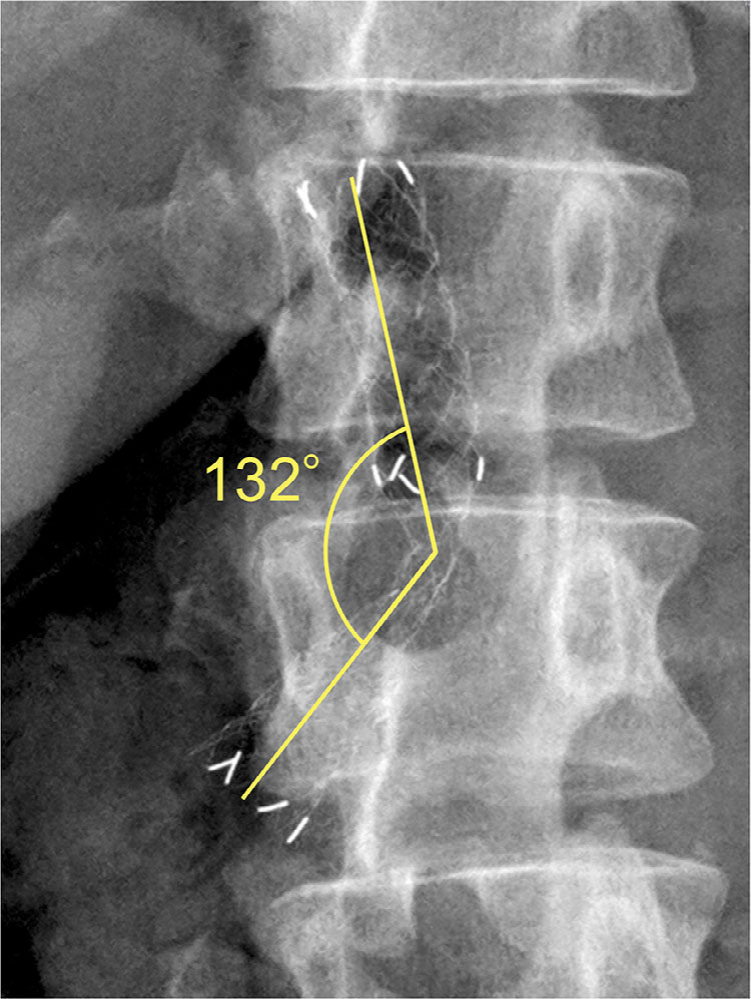
Measurement of the angle of self‐expandable metallic stents (SEMS). The lines extending from the proximal and distal points to the narrowest point were used to measure the obtuse angle of the SEMS, which was 132° after placement in this case.

The primary outcome was the clinical success of the long‐anchoring stent method as a rescue strategy for patients with clinical failure after initial SEMS placement. Clinical success in all 15 patients was also reported descriptively. Secondary outcomes included technical success, changes in SEMS angulation, and the incidence of adverse events. As an exploratory outcome, time to RBO (TRBO) was evaluated for cases that developed early RBO within 90 days of initial SEMS placement. The definitions of clinical success, technical success, RBO, and TRBO were based on the Tokyo Criteria 2024 [17]. Clinical failure was defined as the failure to achieve clinical success after stent placement. For this analysis, TRBO for the initial SEMS was defined as the interval from the initial SEMS placement to RBO diagnosis. TRBO after the anchoring procedure was calculated from the day of anchoring plastic stent placement to the next RBO diagnosis (Figure 2). SEMS angulation was retrospectively measured according to the method reported by Tanoue et al. [18]. Measurements were performed using standing abdominal radiographs obtained on or after the day following SEMS placement. The SEMS angle was defined as the angle formed by lines extending from the proximal and distal ends to the narrowest point (Figure 3).

### 2.4 Statistical Analysis

Continuous variables were compared using the Wilcoxon signed‐rank test. TRBO was evaluated using the Kaplan–Meier method, and differences in TRBO between the initial placement and anchoring procedures in each patient were compared using a stratified log‐rank test as an exploratory analysis. Technical and clinical success were summarized descriptively as absolute numbers without statistical testing. A *p*‐value < 0.05 indicated statistical significance. All statistical analyses were performed using EZR [19] (Saitama Medical Center, Jichi Medical University, Saitama, Japan), a graphical user interface for R (R Foundation for Statistical Computing, version 4.5.2).

## 3 Results

### 3.1 Study Population and Initial Stent Placement

During the study period, 15 patients underwent the long‐anchoring stent method to manage early SEMS dysfunction. Table 1 shows their baseline characteristics. The median age was 61 years (range, 42–85 years); 10 patients were male. The etiologies of distal MBO included pancreatic cancer (*n* = 12), distal cholangiocarcinoma (*n* = 2), and ampullary carcinoma (*n* = 1). Although duodenal invasion was present in 10 patients, none required duodenal stent placement at the time of initial SEMS placement or the subsequent anchoring procedure. Four patients needed duodenal stent placement during their clinical course, with all cases following the RBO of the SEMS with an anchoring stent. A covered SEMS with a diameter of 10 mm and a length ranging from 6 to 8 cm was used for the initial placement.

**TABLE 1:** Patient and procedural characteristics at initial self‐expanding metal stent placement.

	*n* = 15
Age (years), median (range)	61 (42‒85)
Sex, *n* (%)	
Male	10 (66.7)
Female	5 (33.3)
Performance status, *n* (%)	
0	13 (86.7)
1	2 (13.3)
Cancer type, *n* (%)	
Pancreatic	12 (80.0)
Bile duct	2 (13.3)
Ampulla of Vater	1 (6.7)
Chemotherapy, *n* (%)	14 (93.3)
Duodenal invasion, *n* (%)	
No invasion	5 (33.3)
Oral side	1 (6.7)
Papilla level	6 (40.0)
Anal side	3 (20.0)
Prior endoscopic biliary drainage, *n* (%)	
Yes	13 (86.7)
No	2 (13.3)
Endoscopic sphincterotomy, *n* (%)	
Yes	4 (26.7)
No	11 (73.3)
Initial SEMS, *n* (%)	
Niti‐S	7 (46.7)
BONASTENT	5 (33.3)
HANARO STENT	3 (20.0)
Type of initial SEMS, *n* (%)	
Fully covered	14 (93.3)
Partially covered	1 (6.7)
Diameter of SEMS (mm), *n* (%)	
10	15 (100)
Length of SEMS (cm), *n* (%)	
6	4 (26.7)
7	7 (46.7)
8	4 (26.7)

Abbreviation: SEMS, self‐expandable metal stents.

### 3.2 Anchoring Plastic Stent Placement

Anchoring plastic stent placement was performed in five patients with clinical failure and 10 patients with early RBO (within 90 days). In four cases, the initial SEMS was replaced, followed by the insertion of an anchoring plastic stent. In the remaining 11 patients, an anchoring plastic stent was inserted into the existing SEMS after initial SEMS cleaning. Details of the stents placed during the anchoring procedure are summarized in Table 2. A 7‐Fr double‐pigtail plastic stent with a length of 8–15 cm was used as the anchoring stent. Unlike conventional anchoring plastic stent methods [20], relatively longer plastic stents were preferred for this long‐anchoring stent method, with a length of 12 cm being the most frequently used. The most common intrahepatic position of the anchoring stent was the left intrahepatic bile duct (*n* = 9). For duodenal placement, the descending part was observed in nine cases and the horizontal part in six.

**TABLE 2:** Procedural characteristics of the long‐anchoring stent method.

	*n* = 15
Management of initial SEMS, *n* (%)	
Replacement	4 (26.7)
Intraluminal cleaning	11 (73.3)
SEMS type, *n* (%)	
Niti‐S	6 (40.0)
BONASTENT	4 (26.7)
HANARO STENT	4 (26.7)
WallFlex	1 (6.7)
Type of SEMS, *n* (%)	
Fully covered	13 (86.7)
Partially covered	2 (13.3)
Diameter of SEMS (mm), *n* (%)	
10	15 (100)
Length of SEMS (cm), *n* (%)	
6	5 (33.3)
7	6 (40.0)
8	4 (26.7)
Diameter of anchoring plastic stent (Fr), *n* (%)	
7	15 (100)
Length of anchoring plastic stent (cm), *n* (%)	
8	2 (13.3)
10	4 (26.7)
12	8 (53.3)
15	1 (6.7)
Intrahepatic location of plastic stent, *n* (%)	
Common bile duct	4 (26.7)
Left intrahepatic bile duct	9 (60.0)
Right intrahepatic bile duct	2 (13.3)
Duodenal location of plastic stent, *n* (%)	
Descending part	9 (60.0)
Horizontal part	6 (40.0)

Abbreviation: SEMS, self‐expandable metal stents.

### 3.3 Clinical Outcomes of the Long‐anchoring Stent Method

The median follow‐up period after long anchoring stent placement was 307 days (range, 26–974). Regarding the primary outcome, clinical success was achieved in all five patients with initial clinical failure (Table 3). Furthermore, clinical success was achieved in all 15 patients overall, including the 10 patients who experienced early RBO. Technical success rates were comparable between initial SEMS placement and the subsequent anchoring procedure. Regarding the causes of stent dysfunction across all 15 cases, the most frequent causes of stent dysfunction in the initial SEMS group were sludge formation or food impaction (*n* = 14), whereas those in the long‐anchoring stent group included sludge formation or food impaction (*n* = 8), distal migration (*n* = 2), and tumor overgrowth (*n* = 1).

**TABLE 3:** Clinical outcomes of initial self‐expandable metal stents (SEMS) placement and the long‐anchoring stent method for all 15 patients.

	Initial SEMS placement	Long‐anchoring stent method	*p*‐value
Technical success	15/15	15/15	—
Clinical success	10/15	15/15	—
Angle of SEMS (°), median (range)	128.3 (90.9‒172.9)	151.5 (134.2‒178.4)	<0.001
Cause of SEMS dysfunction, *n* (%)			
Sludge formation or food impaction	14 (93.3)	8 (53.3)	
Distal migration	0	2 (13.3)	
Kinking	1 (6.7)	0	
Overgrowth	0	1 (6.7)	
Procedure complications, *n* (%)			
Hyperamylasemia	1 (6.7)	1 (6.7)	
Pancreatitis	1 (6.7)	0	
Cholangitis	1 (6.7)	0	

The en dash (–) indicates that no statistical comparison was performed.

Abbreviation: SEMS, self‐expandable metal stents.

### 3.4 Changes in SEMS Angulation

For all 15 cases, SEMS angulation was significantly corrected from 128.3° (range, 90.9–172.9) with SEMS alone to 151.5° (range, 134.2–178.4) after anchoring stent placement (*p* < 0.001) (Table 3). Detailed information is summarized in Table S1. Regarding anchoring stent position, cases with the distal end in the horizontal section (*n* = 6) showed a trend toward greater correction of SEMS angulation than those with the distal end in the descending section (*n* = 9) (median, 29.7° vs. 16.7°; *p* = 0.088) (Table S2).

### 3.5 Time to RBO

**FIGURE 4:**
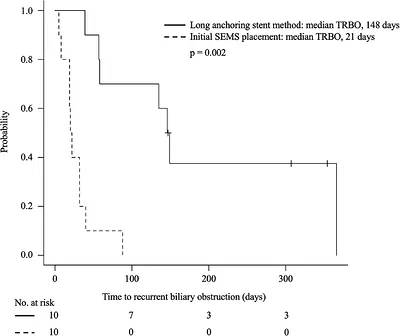
Kaplan–Meier curve (exploratory analysis) comparing time to recurrent biliary obstruction between patients with self‐expandable metallic stent placement alone and those with the long‐anchoring stent method. This cohort includes the 10 patients who achieved clinical success with initial SEMS placement. SEMS, self‐expandable metal stent; CI, confidence interval.

As an exploratory outcome, TRBO improvement was analyzed in the 10 patients who developed early RBO and underwent the long‐anchoring stent method. Among them, RBO was observed in seven patients after the addition of anchoring stents. In these 10 patients, median TRBO was 21 days (95% confidence interval [CI]: 5–32) with the initial SEMS placement alone and 148 days (95% CI: 39–NE) after the long‐anchoring stent procedure (*p* = 0.002) (Figure 4). For all 15 patients, the time to stent dysfunction after the long‐anchoring stent method is shown in Figure S1 (median, 142 days; 95% CI: 58–366).

## 4 Discussion

Covered SEMS are widely used for endoscopic biliary drainage in patients with distal MBO because of their long patency [21] and the option for removal or exchange [22]. Despite their effectiveness, preventing stent dysfunction remains a significant clinical challenge, and various reintervention strategies have been explored to prolong subsequent patency in patients with RBO [23].

This study suggests that the long‐anchoring stent method may be a feasible rescue treatment for achieving effective drainage in patients with early stent dysfunction after initial SEMS placement. Clinical success was achieved in all 15 patients, including in all five patients with initial clinical failure, with a favorable safety profile.

The primary clinical advantage of this method, which may have contributed to these favorable outcomes, is its effectiveness in straightening angulated SEMS. Severe angulation of the SEMS impairs bile flow, leading to accelerated sludge formation [11]. Tanoue et al. reported that sharp SEMS angulation, particularly at an angle <130°, is a significant risk factor for RBO [18]. Our within‐patient observations are consistent with these findings. The median angle of the initial SEMS in our cohort was 128.3°, indicating a relatively severe angulation. However, following placement of the long‐anchoring stent, the SEMS angle significantly improved to 151.5°, indicating the effectiveness of our method in correcting severe SEMS angulation. Moreover, our results suggest that straightening the SEMS may be an important mechanism for improving biliary drainage and preventing early stent dysfunction. These results suggest the clinical utility of this method as a viable and safe re‐intervention strategy for patients who develop early stent dysfunction after covered SEMS placement.

Duodenal invasion was frequently observed in this study. Duodenal invasion and strictures cause anatomical distortion, which can cause the distal end of the SEMS to orient toward the oral side, leading to more frequent food impaction and duodenal content reflux [24]. Our long‐anchoring stent method directs the distal end of the SEMS toward the anal side (Figure 1), reducing the reflux of duodenal contents into the SEMS as well as obstruction due to food impaction. This mechanism suggests that the long‐anchoring method may be effective for both cases with severe SEMS angulation and those with poor biliary drainage due to duodenal invasion.

Conventionally, the “anchoring stent method,” which involves inserting double‐pigtail plastic stents inside a covered SEMS, is reportedly effective in preventing stent migration [20]. In this traditional method, the primary aim of anchoring is to fix the SEMS position. Therefore, the distal ends of plastic stents are typically positioned at the same level as the distal end of the SEMS, applying a traction force toward the hepatic side to prevent distal migration. Conversely, our novel method differs fundamentally in its purpose. It is designed not to fix the SEMS position, but to optimize biliary drainage. This method aims to straighten the angulated SEMS by reinforcing the axial force and directing the distal end of the SEMS toward the anal side using a long plastic stent. To achieve these objectives, the choice of a “long” stent is critical. Placing a long stent provides the force necessary to direct the distal end of the SEMS toward the anal side. Further, we observed a trend toward greater SEMS straightening in cases in which the anchoring stent reached the horizontal portion, which may indicate the potential benefit of applying force with a longer stent. This technique was designated as the “long”‐anchoring stent method, in which a long plastic stent facilitates the ideal SEMS shape and orientation. Regarding the proximal end, a straightening effect is consistently observed whether the stent is positioned in the common bile duct or intrahepatic bile ducts.

Recently, endoscopic ultrasound (EUS)‐guided biliary drainage, specifically EUS‐guided hepaticogastrostomy (EUS‐HGS), has emerged as a potential option for the management of early RBO or distal MBO with duodenal invasion [25, 26]. Draining bile directly into the stomach carries a lower risk of duodenal content reflux, potentially reducing the risk of early RBO [27]. However, EUS‐HGS involves a risk of complications, such as bile peritonitis, stent migration, and hemorrhage [28]. Transpapillary procedures, including our long‐anchoring stent method, may be preferable for patients with a high risk of EUS‐HGS procedure‐related complications, such as hemorrhage or ascites. Conversely, EUS‐HGS may be more suitable for patients with severe duodenal strictures or for those for whom transpapillary drainage is technically challenging. Similarly, duckbill‐type anti‐reflux SEMS have demonstrated prolonged patency in reintervention settings [29, 30]. These stents are effective, even in patients with duodenal strictures, because the anti‐reflux valve prevents reflux into the bile duct [31]. Conversely, some reports indicate difficulty in removing these stents upon occlusion, which may complicate subsequent reinterventions [32]. Ultimately, although multiple drainage techniques have been developed for patients who fail conventional transpapillary stenting, the optimal approach for reintervention should be individualized according to the clinical context [33].

Clinically, applying the long‐anchoring stent method to all cases may not be practical owing to cost and technical complexity. This method should be selectively used for patients with severe SEMS angulation due to malignancy or those with anatomical features likely to cause food impaction. Notably, our study targeted patients with clinical failure or early RBO after the initial SEMS placement to evaluate drainage efficacy while reducing potential confounding factors, such as tumor progression. In these cases, with early stent dysfunction, the SEMS might have been more flexible, making it easier to change the stent angle or achieve a straightening effect with the anchoring stent. Therefore, further investigation is needed to determine whether the same mechanical effects and prolonged TRBO can be achieved in patients who develop RBO after >90 days post SEMS placement. Additionally, even during initial stenting, cases with a highly angulated bile duct or duodenal invasion are at high‐risk of RBO. Therefore, preprocedural evaluation of the bile duct axis using axial and coronal views of contrast‐enhanced computed tomography, and intraprocedural endoscopic or cholangiographic findings during ERCP are crucial for identifying these high‐risk features. Our method may be effective in cases where these high‐risk conditions are suspected during initial SEMS placement. However, since completely predicting the patency duration is difficult based only on imaging findings, the role of this method in initial stenting remains unclear and warrants future investigation for these high‐risk cohorts.

This study has some limitations. First, it was conducted at a single center with a small sample size; therefore, accurately evaluating the clinical utility of this method within a single‐arm retrospective study could be difficult. Second, the decision to apply the long‐anchoring method was left to the discretion of the endoscopist, which may have introduced a selection bias. Third, the initial SEMS was cleaned or replaced before anchoring stent placement; therefore, the improved drainage may have been partly attributable to removal of the obstructive cause independent of the long‐anchoring method. Fourth, inserting a plastic stent into a poorly draining SEMS can improve drainage and prolong TRBO by itself. Consequently, the specific impact of this technique on clinical outcomes should be interpreted with caution.

In conclusion, the long‐anchoring stent method can be a simple, safe, and viable rescue treatment option for achieving efficient biliary drainage in patients with early dysfunction of a covered SEMS, especially in those with severe SEMS angulation.

## Author Contributions

**Takumi Kinomoto**: contributed to study conceptualization, data curation, formal analysis, investigation, methodology, project administration, visualization, and writing of the original draft. **Yugo Kai**: contributed to study conceptualization, data curation, formal analysis, investigation, methodology, project administration, resources, and writing of the original draft. **Minoru Shigekawa**: contributed to investigation, methodology, project administration, and writing – review and editing. **Kenji Ikezawa**: and **Takuo Yamai**: contributed to study conceptualization, investigation, methodology, project administration, and writing – review and editing. **Hiroki Takiyama, Taku Miyanaga, Takanori Masumoto, Keita Sekiya, Jun Murata, Makiko Urabe, Ryoji Takada, Kaori Mukai**, and **Tasuku Nakabori**: contributed to the investigation and writing – review and editing. **Kazuyoshi Ohkawa**: contributed to the investigation, supervision, and writing – review and editing. All authors contributed to data interpretation, critically reviewed the manuscript, and approved the final version for submission.

## Funding

The authors have nothing to report. None.

## Ethics Statement

This study was approved by the Institutional Review Board of the Osaka International Cancer Institute (Approval No. 18050–9) and was designed and executed in accordance with the ethical standards of the 1964 Declaration of Helsinki and its later amendments.

## Consent

The requirement for informed consent was waived by using the opt‐out method on our hospital's website.

## Conflicts of Interest

Yugo Kai reports honoraria from Kaneka Medix, Piolax Medical Devices, and Medico's Hirata. Kenji Ikezawa reports honoraria from Piolax Medical Devices, J‐MIT INC, and Medico's Hirata. Ryoji Takada reports honoraria from AstraZeneca, Merck, and Novartis. Minoru Shigekawa reports honoraria from Kaneka Medix, Boston Scientific, J‐MIT INC, Asahi Intecc, Medico's Hirata, and Gaderius Medical. K.O. reports honoraria from Chugai Pharmaceutical, Eisai, AstraZeneca, and Incyte.

## Use of Generative Artificial Intelligence

ChatGPT (OpenAI) and Gemini (Google) were used solely for language editing under the supervision of the authors. No AI was used in data generation, analysis, or interpretation.

## Supporting information




**FIGURE S1**: Kaplan–Meier curve of time to stent dysfunction with the long‐anchoring stent method. This cohort includes all 15 patients who received a long‐anchoring stent method as a reintervention. The median time to stent dysfunction was 142 days (95% CI: 58–366). SEMS, self‐expandable metal stent.

**TABLE S1**: Patients who underwent the long‐anchoring stent method. SEMS, Self‐expandable metal stents; TRBO, time to recurrent biliary obstruction.
**TABLE S2**: Stent selection and SEMS angle changes according to the duodenal location of anchoring stents. SEMS, self‐expandable metal stents.

## Data Availability

The data that support the findings of this study are not openly available due to reasons of sensitivity and are available from the corresponding author upon reasonable request. Data are located in controlled access data storage at Osaka International Cancer Institute.
