# Supplementary material for: Long‐anchoring Stent Method as a Rescue Technique for Early Dysfunction of Covered Self‐Expandable Metal Stents in Distal Malignant Biliary Obstruction: A Retrospective Case Series
Source: DEN Open. 2026 Sep 3;7(1):e70394. doi: 10.1002/deo2.70394 (PMC13540643; doi:10.1002/deo2.70394)
Supplement: Supplementary file 1 [file DEO2-7-e70394-s001.docx]

|  | | Supplementary Table 1. Patients who underwent the long-anchoring stent method | | | | | | | | | | | |
| --- | --- | --- | --- | --- | --- | --- | --- | --- | --- | --- | --- | --- | --- |
| Patients | Cancer type | | SEMS used for the long-anchoring stent method (mm) | Duodenal invasion | Length of the anchoring plastic stent (cm) | Intrahepatic location of the plastic stent | Duodenal location of the plastic stent | Angle of the SEMS at the initial SEMS placement (°) | Angle of the SEMS at the long-anchoring stent method (°) | Change in the SEMS angulation (°) | Time from initial SEMS placement to dysfunction (days) | TRBO of the long anchoring stent method (days) |  |
| 1 | Bile duct | | WallFlex, 10 × 70 | – | 12 | Common bile duct | Descending part | 131.7 | 151.8 | +20.1 | 2  (Clinical failure) | 138 |  |
| 2 | Pancreatic | | Niti-S ComVi stent, 10 × 60 | – | 10 | Left intrahepatic bile duct | Descending part | 128.3 | 151.5 | +23.2 | 20 | 146 |  |
| 3 | Bile duct | | BONASTENT, 10 × 80 | Papilla level | 12 | Left intrahepatic bile duct | Descending part | 166.1 | 178.4 | +12.3 | 8 | 147  (Died without RBO) |  |
| 4 | Pancreatic | | Niti-S ComVi stent, 10 × 60 | Anal side | 12 | Common bile duct | Descending part | 122.0 | 143.5 | +21.5 | 19 | 307  (Died without RBO) |  |
| 5 | Pancreatic | | Niti-S ComVi stent, 10 × 80 | Papilla level | 10 | Right intrahepatic bile duct | Descending part | 127.0 | 134.2 | +7.2 | 19 | 58 |  |
| 6 | Pancreatic | | BONASTENT, 10 × 60 | – | 15 | Left intrahepatic bile duct | Horizontal part | 102.7 | 145.3 | +42.6 | 4  (Clinical failure) | 420 |  |
| 7 | Ampulla of Vater | | HANARO Stent, 10 × 70 | Papilla level | 8 | Left intrahepatic bile duct | Descending part | 146.5 | 151.2 | +4.7 | 32 | 39 |  |
| 8 | Pancreatic | | HANARO Stent, 10 × 70 | Papilla level | 12 | Common bile duct | Horizontal part | 118.5 | 139.8 | +21.3 | 7  (Clinical failure) | 99 |  |
| 9 | Pancreatic | | HANARO Stent, 10 × 60 | Papilla level | 12 | Left intrahepatic bile duct | Horizontal part | 132.3 | 157.3 | +25.0 | 88 | 366 |  |
| 10 | Pancreatic | | HANARO Stent, 10 × 70 | Anal side | 12 | Left intrahepatic bile duct | Horizontal part | 172.9 | 174.7 | +1.8 | 22 | 149 |  |
| 11 | Pancreatic | | Niti-S ComVi stent, 10 × 80 | Anal side | 12 | Common bile duct | Horizontal part | 119.0 | 153.3 | +34.3 | 6  (Clinical failure) | 100 |  |
| 12 | Pancreatic | | BONASTENT, 10 × 70 | Papilla level | 10 | Left intrahepatic bile duct | Descending part | 171.5 | 178.4 | +6.9 | 5 | 354  (Died without RBO) |  |
| 13 | Pancreatic | | Niti-S ComVi stent, 10 × 70 | – | 10 | Right intrahepatic bile duct | Descending part | 127.2 | 143.9 | +16.7 | 6  (Clinical failure) | 26  (Died without RBO) |  |
| 14 | Pancreatic | | Niti-S ComVi stent, 10 × 80 | – | 12 | Left intrahepatic bile duct | Horizontal part | 90.9 | 145.9 | +55.0 | 32 | 135 |  |
| 15 | Pancreatic | | BONASTENT, 10 × 60 | Oral side | 8 | Left intrahepatic bile duct | Descending part | 151.2 | 176.0 | +24.8 | 40 | 57 |  |

| Supplementary Table 2. Stent selection and SEMS angle changes according to the duodenal location of the anchoring stent | | | | | | | | | | |  |
| --- | --- | --- | --- | --- | --- | --- | --- | --- | --- | --- | --- |
|  |  |  |  |  |  |  |  |  |  |  |  |
|  |  |  |  |  |  |  |  | | |  |  |
|  |  |  | Descending part (n=9) |  | Horizontal part (n=6) |  | P value | | |  |  |
|  | Length of the anchoring plastic stent (cm) |  | 10 (8‒12) |  | 12 (12‒15) |  | 0.014 | | |  |  |
|  | SEMS angle change induced by the anchoring stent method (°) |  | 16.7 (4.7‒24.8) |  | 29.7 (1.8‒55.0) |  | 0.088 | | |  |  |
| Continuous variables are presented as median (range).  SEMS, Self-expandable metal stents. | | | | | | | |  |  | |  |
|  |  |  |  |  |  |  |  |  |  | |  |
